# Dual-Channel Voice Communication System Based on One-Way Quantum Secure Direct Communication—Classical Optical Communication Hybrid Mode

**DOI:** 10.3390/e28060707

**Published:** 2026-06-18

**Authors:** Xiuwei Chen, Dong Pan, Jianxing Guo

**Affiliations:** Beijing Academy of Quantum Information Sciences, Beijing 100193, China

**Keywords:** unidirectional quantum secure direct communication, duplex voice communication, one-time pad

## Abstract

Quantum secure direct communication, as an important branch of quantum communication, possesses strict information-theoretic security and can achieve secure communication in channel environments with noise interference and eavesdropping threats. As voice communication is the most fundamental and widespread communication method in daily life, guaranteeing its security and efficiency has become an important research topic in current communication technology. One-way quantum secure direct communication technology can build an efficient and reliable security barrier for voice communication services, effectively preventing the leakage of private information in voice communication. This paper proposes a duplex voice communication scheme based on one-way quantum secure direct communication. By adopting a method combining multi-task parallel processing and stream processing, the communication rate and transmission delay performance of the system are significantly improved. Relying on quantum secure direct communication technology and the one-time-key encryption channel within the system, duplex voice communication is achieved securely. The real-time temperature drift compensation algorithm is introduced to ensure the long-term stable operation of the system. At the same time, through the real-time temperature drift prediction mechanism, the strategy selection during the call process is optimized to ensure the quality of the voice communication. To verify the feasibility and performance of this scheme, a one-way quantum secure direct communication duplex voice communication system was built in the laboratory environment, and comprehensive performance indicator tests were conducted. The test results show that the constructed one-way quantum secure direct communication system can fully meet the performance requirements of duplex voice communication. The realization of this system successfully achieves the goal of secure and efficient quantum voice communication, laying an important technical foundation for further expanding the practical application scenarios of quantum communication technology and promoting the industrialization development of quantum communication.

## 1. Introduction

Voice communication is widely applied in all fields of modern social economic life, covering various industries. Due to its core advantages of real-time communication and interactivity, it has also been widely used in industries with extremely high requirements for communication security, such as military, government, and finance. Currently, a large amount of sensitive voice data is transmitted through various methods in different voice communication systems across various industries. Attackers can easily obtain data containing sensitive information through eavesdropping technology, thereby triggering potential economic risks, defense security information leakage, and other problems. Therefore, ensuring the security of voice calls has become a research focus in the field of communication.

Quantum secure direct communication (QSDC) transmits information through quantum states and has strict information-theoretic security. It has developed rapidly in recent years and relevant methods and solutions for practicality, security, and high transmission rate have been proposed and continuously improved [1,2,3,4,5,6,7,8,9,10,11,12,13,14]. In 2019, the world’s first quantum secure direct communication principle prototype was released in Beijing, marking the entry of quantum secure direct communication technology into a substantive development stage. Related technical achievements have continued to emerge [5,6,9,10]. Currently, the communication distance of one-way quantum secure direct communication systems can reach over 100 km [12,14], and all system indicators can meet the requirements of actual application scenarios. As a key component of wide-area quantum networks, free-space QSDC has been verified in laboratory environments and short-range field tests (e.g., urban links spanning several kilometers) [13], laying a foundation for the future development of satellite–ground quantum direct communication.

The architecture of a one-way quantum secure direct communication system consists of a quantum channel and an auxiliary classical channel. The quantum channel uses quantum states as the information carrier and can directly achieve secure information transmission, forming a completely new communication paradigm. This paradigm expands reliable communication under noisy channels from traditional classical communication to reliable and secure communication under noisy and eavesdropping coexisting channels, further enhancing the security level of the communication system.

The proposed solution in this paper utilizes the high bandwidth advantage of the auxiliary classical channel and combines the keys generated by the one-way quantum secure direct communication system to achieve one-time encryption transmission of voice data [15,16]. This forms a duplex voice communication architecture within the one-way quantum secure direct communication system, enabling secure duplex transmission of voice data, and creates a voice communication service solution that is both secure and efficient. This solution’s technology can effectively enhance the confidentiality of traditional voice communication. Its implementation and feasibility tests have significant practical significance and application value in ensuring the security of important information data related to national economy and people’s livelihood and preventing information leakage.

## 2. System and Link Initialization Method

**Figure 1:**
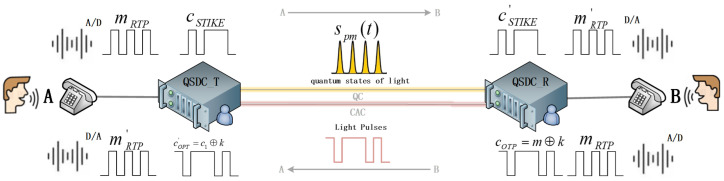
The composition of a one-way quantum secure direct communication duplex voice system. QC, quantum channel; CAC, classic auxiliary channel.

Figure 1 shows the composition architecture of the quantum voice duplex communication system. This system is mainly composed of telephone terminal equipment and a one-way quantum secure direct communication system device. Its architecture conforms to the ISO/OSI model. Within the system, voice communication over VoIP adopts the Session Initiation Protocol (SIP) The one-way quantum secure direct communication system includes a quantum channel and a classical auxiliary channel. The two channels are independently realized through two optical fiber lines, respectively: the quantum channel is responsible for completing the secure voice communication from the A end to the B end, while the classical auxiliary channel uses a gigabit Ethernet optical fiber channel, undertaking one-time one-key encryption voice communication from the B end to the A end. The collaborative work of the two channels realizes duplex voice security communication.

During the voice data transmission from the A end to the B end, the process is as follows: First, the analog voice signal is converted into a digital signal. Then, the voice data is encapsulated into a Real-Time Transport Protocol (RTP) data packet according to relevant protocols. In Figure 1, m_RTP_ refers to the digital pulse signal obtained by modulating the data packets encapsulated under the RTP. **c_STIKE_** represents the digital signal generated after encoding by the transmitter side of the STIKE protocol [14]. Next, the voice RTP data packet is injected into the one-way quantum communication system. The system performs processing such as channel forward error correction coding, spread spectrum, encryption, mask expansion, and quantum state preparation phase encoding on the data in sequence according to the STIKE protocol [14], and modulates the encoded bit stream data using the decoy state method. Finally, the generated quantum state signal completes the information transmission through the quantum channel. At the quantum secure direct communication (QSDC) receiving end, the quantum state signal is also demodulated and detected, demasking processing, and decoded, and security identification and judgment are carried out using a series of processing such as the STIKE protocol, restoring the voice RTP data packet, thereby achieving voice transmission and playback from the A end to the B end.

During the voice data transmission from B to A, the process is basically the same as that from A to B: the analog voice signal is first converted into a digital signal, and then encapsulated into RTP data packets according to the protocol; subsequently, the voice RTP data packets undergo one-time-key encryption processing in the unidirectional quantum communication system by using the security key generated by the system itself; the encrypted voice data packets are transmitted through the classical auxiliary channel, and the receiving end completes decryption and data restoration operations to achieve voice transmission and playback from B to A. Among them, the security key data is generated by the post-processing module in the STIKE protocol of the unidirectional quantum secure direct communication system [14].

**Figure 2:**
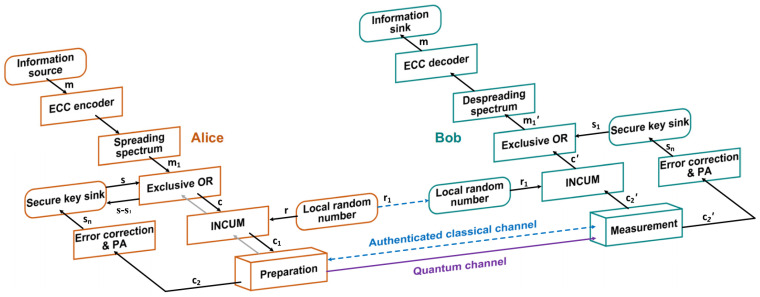
Schematic of one-way quasi-QSDC [14]. ECC, error correction code; INCUM, increase capacity using masking; PA, privacy amplification.

Figure 2 presents the STIKE protocol, which mainly consists of core functions such as preprocessing, preparation and measurement, as well as post-processing. Its implementation process is completed through 12 processing steps [14]. Herein, **m** represents the source data at the transmitter of the quantum communication system; **c** denotes the data obtained after channel encoding of the source data and XOR processing with the security sequence via the STIKE protocol; **r** refers to the local random number data processed by the INCUM module of the STIKE protocol.

Specifically, the formatted message **m** is generated by processing the original message **m** via a cascaded encoding module composed of an error correction code (ECC) encoder and a spread-spectrum encoder. Subsequently, the derived **m** is encrypted through a bitwise exclusive OR (XOR) operation with a key segment s of identical length. Notably, the key segment **s** for the encryption of **m** serves as a subset of the pre-shared key **s**, which is synchronously stored in the secure key reservoirs of both communication parties (Alice and Bob). According to the index data extracted from detected signal states, Bob is capable of locating and acquiring the matched **s** from the local key repository.

For the STIKE protocol proposed in the study Reference [14], functional integration of information transmission and key negotiation is realized in a single communication round. Different from conventional negotiation schemes, the information transmission process of this protocol adopts pre-stored keys from the existing key pool for encryption, instead of the fresh keys generated in the ongoing key negotiation phase. For this reason, the adopted segment **s** in Bob’s terminal is not a subset of the newly generated key sequence **s**. The key sequence **s** is exclusively reserved for subsequent communication scenarios and is not activated for the current data transmission session. Consequently, the **s_1_** that Bob uses to recover **m_1_′** has not undergone privacy amplification; the quantum secure direct communication of information **m** is finally accomplished.

**Figure 3:**
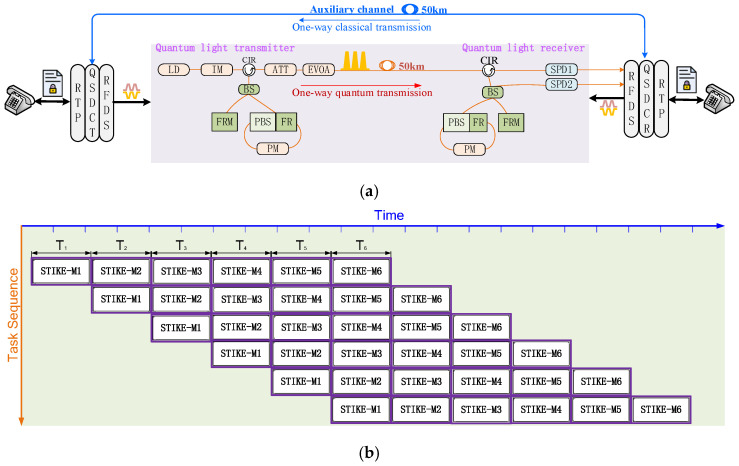
The optical path and protocol composition of the system. (**a**) Deployment of the STIKE protocol and realization of the optical path system; (**b**) STIKE protocol—multi-task parallel processing. LD: laser diode, IM: intensity modulator, BS: beam splitter, PBS: polarization beam splitter, FM: Faraday mirror, FR: Faraday rotator, PM: phase modulator, CIR: optical circulator, VOA: variable optical attenuator, SM: single mode, SPD: single-photon detector, QSDCT: Quantum Secure Direct Communication Transmitter, QSDCR: Quantum Secure Direct Communication Receiver, RTP: Real-Time Transport Protocol.

Figure 3a illustrates the deployment of quantum optical units and security protocol modules for the duplex system of QSDC nodes within the unidirectional quantum secure direct communication system. The sending end of the node system includes RTP (Real-Time Transport Protocol), the sending QSDCT (Quantum Secure Direct Communication Transmitter) protocol module, and RFDS (Radio Frequency Digital Signal) radio frequency modulation and demodulation modules, etc. The receiving end of the node system is equipped with RTP, the receiving QSDCR (Quantum Secure Direct Communication Receiver) protocol module, and RFDS (Radio Frequency Digital Signal) radio frequency modulation and demodulation modules, etc. The sending end and the receiving end QSDC use the STIKE protocol to achieve secure transmission of important data between the two network element servers. The STIKE protocol is structured into 6 functional processing modules based on the system composition and resource allocation. The specific modules include: (1) key management, channel coding, and phase encoding; (2) error correction and secure amplification, random data encoding, and PCIE (Peripheral Component Interconnect Express) interface data transmission; (3) quantum state preparation and transmission, quantum state detection and analysis; (4) detection data encapsulation and PCIE interface data transmission; (5) auxiliary information disclosure, basis vector comparison, channel decoding, and error correction and secure amplification; (6) cipher text data restoration and synthesis, and key management. To optimize the system’s communication rate and delay performance, this scheme adopts the multi-threaded pipeline processing architecture as shown in Figure 3b, and introduces a multi-threaded parallel processing mechanism in the channel decoding stage to effectively reduce processing delay. Among them, STIKE-M1 to STIKE-M6 correspond successively to the 6 functional processing modules (1) to (6) mentioned above.

In Figure 3b, the first pipeline corresponds to the execution process of the six functional modules of the STIKE protocol. The single transmission delay of the system is the sum of the processing times of the six functional modules of the STIKE protocol. The sum of processing times is expressed as

(1) $$T_{\mathrm{delay}}=T_{1}+T_{2}+T_{3}+T_{4}+T_{5}+T_{6}$$

If the single quantum secure direct communication does not adopt the multi-task pipelining processing method and the transmitted data volume is *K*, then the quantum secure direct communication rate of this communication method can be expressed as

(2) $$R_{s}=\frac{K}{T_{delay}}$$

Using the 6-level pipelining processing method shown in Figure 3, by reasonably allocating and optimizing computing resources, the 6 functional units can execute in parallel; among them, the processing time of different functional units in each level of the pipeline fluctuates within a certain range; that is,

(3) $$T_{n}\in (T_{MINn},T_{MAXn})$$

When using the multi-task parallel processing method, the communication rate of the system is determined by the processing unit with the longest processing time. Suppose the fluctuation range of the maximum processing time among the 6 processing units is

(4) $$T_{LM}\in (T_{MIN_{LM}},T_{MAX_{LM}})$$

Therefore, in the batch processing mode, the communication rate of the system can be expressed as

(5) $$R_{\mathrm{con}}=\frac{K}{T_{LM}}$$

From the above Equations (1)–(5), it can be seen that the continuous communication rate after pipelining processing is higher than that of non-pipelined processing; if the computing resources are sufficient and the design is reasonable, the communication rate can be maximally improved, while the transmission delay can be reduced, thereby meeting the real-time requirements of voice communication.

In the duplex voice communication architecture based on the one-way quantum secure direct communication system shown in Figure 1, the quantum channel transmission based on the STIKE protocol requires the use of the key data generated by the system to encrypt the information, while the voice data of the classical auxiliary channel needs to be encrypted using the one-time pad encryption method, which requires the key data to be of the same length as the voice data to meet the encryption transmission requirements. To ensure the security and continuity of voice communication, the key generation rate of the one-way quantum secure direct communication system needs to meet the encryption processing requirements of both channels in the duplex voice communication; therefore, the condition equation for ensuring communication security and continuity is

(6) $$C_{SQ}=C_{SQi}+R_{SQ}\times T_{\mathrm{run}}-G_{recycle}\times R_{\mathrm{con}}\times T_{\mathrm{com}}\times K_{\mathrm{cor}}-R_{OPT}\times T_{com}$$

When the key quantity $C_{\mathrm{SQ}}$ is greater than zero, the system can maintain secure duplex communication and can predict the duration of sustainable secure communication. Here, $C_{\mathrm{SQi}}$ represents the initial available key quantity; $R_{\mathrm{SQ}}$ is the rate at which the system generates its own keys; $T_{\mathrm{run}}$ is the running time of the one-way quantum secure direct communication system; $R_{\mathrm{con}}$ is the transmission rate of the quantum channel; $T_{\mathrm{com}}$ is the duration of secure communication; $R_{\mathrm{OPT}}$ is the rate of one-time-keyed voice data transmission over the classical auxiliary channel; $G_{\mathrm{recycle}}$ represents the reception rate of one-way quantum secure direct communication, which is determined by factors such as channel loss, the efficiency of the single-photon detector, and the proportion of public channels revealing the base information, and is determined through measured data during the system operation; $K_{\mathrm{cor}}$ is determined by the forward error correction coding rate of the system channel and the spread-spectrum factor, and its expression is

(7) $$K_{\mathrm{cor}}=KN_{\mathrm{code}}\times K_{SS}$$

$KN_{\mathrm{code}}$ is the reciprocal of the channel coding rate, and $K_{\mathrm{SS}}$ is the spreading length, which are determined by factors such as communication distance, channel attenuation and disturbance parameters.

**Figure 4:**
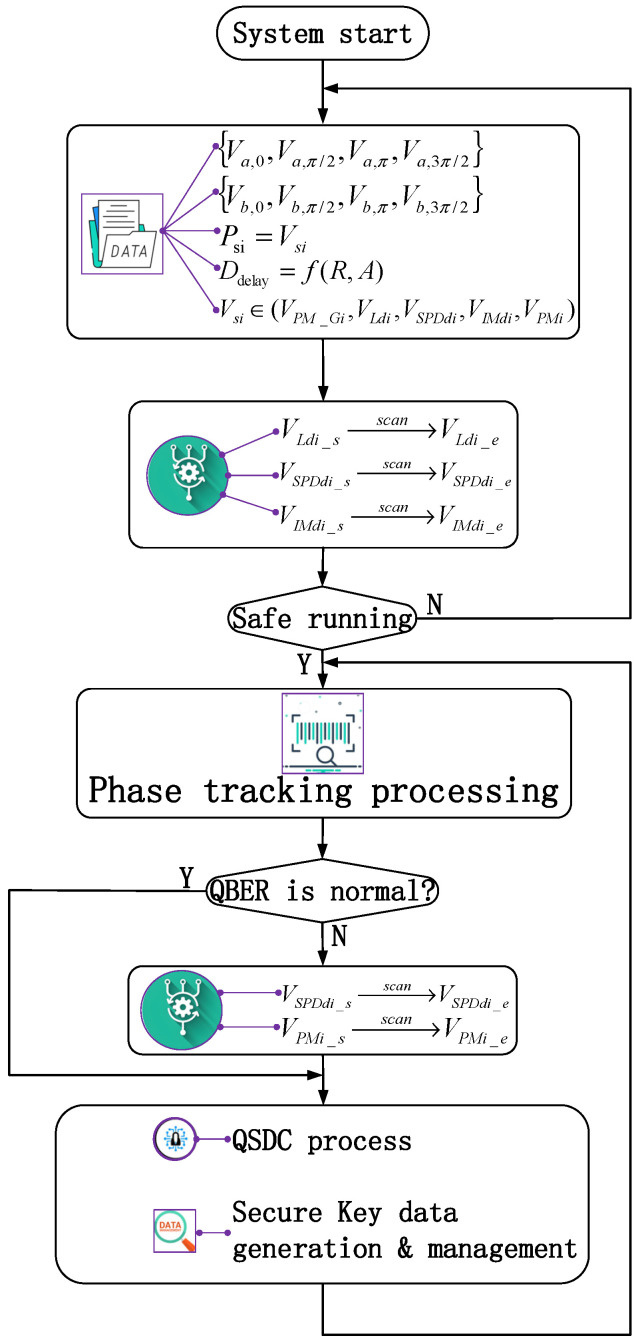
Stable operation processing method for one-way QSDC system.

The method for stabilizing the operation of the one-way quantum secure direct communication system is shown in Figure 4. According to this method, quantum state information transmission through the quantum channel in the duplex voice communication system can be achieved.

As shown in Figure 4, the transmitting end and the receiving end of the one-way quantum secure direct communication system first read the initialization file of the quantum channel parameters. This file contains the relevant parameters of the optical unit of the terminal device. Among them, {*V_a,0_*, *V_a,π/2_*, *V_a,π_*, *V_a,3π/2_*} and {*V_b,0_*, *V_b,π/2_*, *V_b,π_*, *V_b,3π/2_*} are the initialization parameter values of the phase modulators in the optical units of the transmitting end and the receiving end, respectively. These parameters directly determine the four encoding phase modulation voltages of the electrical RF units at the transmitting end and the receiving end of the system; *P_si_* = *V_si_* and *D_delay_* = *f*(*R*,*A*) are the line attenuation estimation value and the delay estimation value, respectively. Among them, *V_si_* ∈ (*V_PM_Gi_, V_Ldi_*, *V_SPDdi_*, *V_IMdi_*, *V_PMi_*); the corresponding parameters are the initial values of the phase modulator gain, the initial value of the laser’s small delay, the initial value of the single-photon detector’s small delay, the initial value of the optical intensity modulator’s small delay, and the initial value of the phase modulator voltage at the transmitting end and the receiving end, respectively; *D_delay_* = *f*(*R*,*A*) is the quantum light transmission delay time of the quantum circuit, and this parameter can further confirm the synchronization delay parameter between the transmitting end and the receiving end, ensuring that the system successfully completes the link initialization and runs stably under the error rate threshold that meets the security requirements [17].

The one-way quantum secure direct communication system conducts parameter scanning based on the above initial parameter values. To minimize the scanning time, the initial scanning adopts the local scanning method to determine the parameter values of the quantum channel link. The local scanning parameters include the small delay of the laser emission, the small delay of the intensity modulator, the small delay of the phase modulator, and the small delay of the single-photon detector. If the parameter values determined by the local scanning can enable the system to operate stably within the safe error rate threshold during quantum transmission, then the phase drift is further corrected by the phase compensation algorithm to maintain the stable operation of the system. In the system, the real-time temperature drift compensation adopts the real-time tracking phase compensation algorithm [18], and the phase compensation formula is as follows:

(8) $$\Delta V_{i}^{\prime }=K_{P}\Delta V_{i}+K_{I}\sum_{n=1}^{i}\Delta V_{n}+K_{D}(\Delta V_{i}-\Delta V_{i-1})$$

(9) $$V_{i+1}=V_{i}+\Delta V_{i}^{\prime }$$

The temperature drift compensation process calculates the corresponding compensation voltage difference for the phase drift through Equation (8), and then calculates the modulation voltage to be output to the phase modulator through Equation (9), thereby completing the real-time active phase compensation process of the quantum channel. The relationship in Equation (10) [19] between the system QBER (quantum bit error rate, QBER) and the phase indicates that phase errors will significantly deteriorate the system QBER, thereby affecting the normal operation of duplex voice communication, while the phase compensation algorithm constructed through Equations (8) and (9) can effectively suppress the adverse effects brought by temperature drift, maintain the normal operation of the quantum channel link of the system, and ensure the stability of duplex voice communication.

(10) QBERphase=sin2(Δϕe2)

## 3. Experimental Results and Performance Analysis

As shown in Figure 1, the repetition frequency of the transmitting pulse in the one-way quantum secure direct communication system is 1.25 GHz, the pulse width of the weak laser pulse is 50 ps, and the pulse intensity is 0.6 photons per pulse; the working wavelength of the weak light pulse is 1550 nm, and the working wavelength of the classical auxiliary channel laser is 1550 nm/1310 nm. The system adopts an optical fiber transmission method, with a transmission distance of 50 km; the optical fiber attenuation index is 0.2 dB/km, and the ambient temperature for system testing is controlled within the range of 24 to 27 °C. This one-way quantum secure direct communication system adopts the AXIe (Advanced TCA Extensions for Instrumentation and Test, AXIe) architecture, with the computer system motherboard equipped with an Intel i5 processor and 32 GB of memory; the main control board card is a PCIe interface board card with a high-speed FPGA as the core, and the computer system completes the execution of the STIKE protocol and other various functional tasks through the multi-task parallel pipelining processing mode.

**Figure 5:**
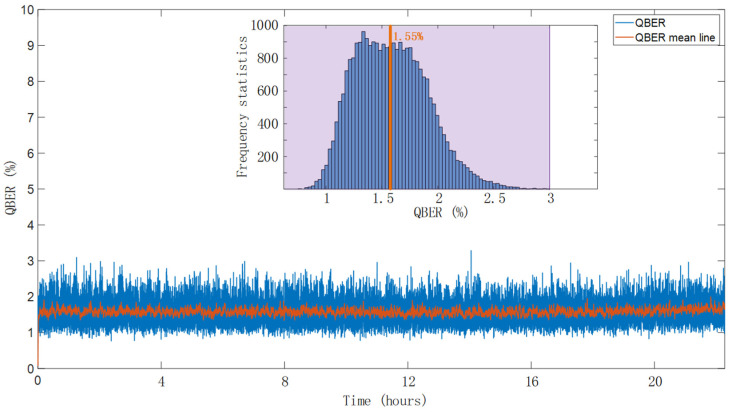
QBER of one-way quantum secure direct communication system.

Figure 5 presents the quantum QBER curve corresponding to the duplex voice communication procedure of the unidirectional quantum secure direct communication system illustrated in Figure 1.

The STIKE protocol adopted in this system generates secure keys based on standard Quantum Key Distribution (QKD). The measurement procedure for QBER is described as follows:

(1) Perform quantum state transmission and random basis measurement over the 1550 nm quantum channel.
(2) Disclose basis information via the 1310 nm classical channel, retain bits with matching bases, and generate the sifted key.
(3) Randomly select 10% of the detected bits for public comparison and count the number of error bits.
(4) Calculate the QBER using the formula

$$QBER=\frac{N_{error}}{N_{sifted}}$$

where N_error_ denotes the number of error bits and N_sifted_ is the total number of sifted bits.

From the test data and histogram statistics results in Figure 5, it can be observed that under the laboratory working conditions, the average value of the system’s quantum bit error rate is approximately 1.55%. By using the system stable operation processing method shown in Figure 5, combined with the temperature drift phase compensation algorithm of Equations (8) and (9), the system can ensure long-term stable operation and successfully realize the duplex voice communication function.

**Figure 6:**
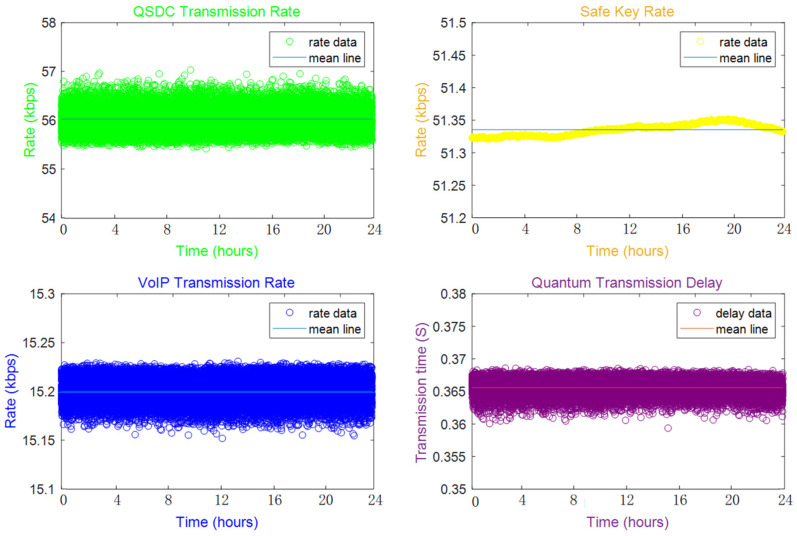
One-way QSDC duplex voice communication system operation indicators.

Figure 6 presents the operational indicators of the unidirectional quantum secure direct communication system in the duplex voice communication mode, mainly including the unidirectional quantum secure direct communication transmission rate, secure key rate, VoIP data packet transmission rate, and transmission delay.

The system adopts the STIKE protocol and performs post-processing of secure keys based [20] on standard Quantum Key Distribution (QKD). The detailed procedures are as follows:

(1) Sifting: Retain bits with matching bases and discard mismatched data.
(2) Parameter Estimation: Sample and calculate the QBER, evaluate channel conditions and eavesdropping status, and set the security threshold.
(3) Error Correction/Reconciliation: Correct key errors via the public classical channel. The system adopts the LDPC algorithm [21] to eliminate errors introduced by the system.
(4) Privacy Amplification [22]: Construct a hash function based on the Toeplitz matrix to compress the keys, remove information obtained by eavesdroppers, and generate the final secure keys.

Secure keys can be extracted after completing the above post-processing procedures. The secure key rate is calculated by counting the volume of secure keys output per unit time.

The measured results show that the average unidirectional quantum secure direct communication transmission rate is approximately 56 kbps, the average secure key rate is approximately 51.3 kbps, and the average VoIP data packet transmission rate is approximately 15.2 kbps. This voice service only occupies a very small bandwidth on the classical auxiliary channel and will not affect the data transmission of the auxiliary information of the STIKE protocol; the average unidirectional quantum secure direct communication transmission delay is approximately 365 ms.

The one-way quantum secure direct communication transmission rate and security key rate are indicators obtained after optimizing and improving through the parallel multi-task processing method of the STIKE protocol as shown in Figure 2 under the condition of limited system computing resources. The VoIP call data packet transmission rate adopts the typical rate of commercial telephones, while with the continuous optimization of the voice compression algorithm, the actual voice data code rate can be further reduced [23,24,25,26]. Therefore, the quantum channel bandwidth of the one-way quantum secure direct communication system supports simultaneous access by multiple telephones, enabling concurrent secure voice communication for multiple user terminal devices, thus adapting to a wider range of application scenario requirements.

During the duplex voice communication process of the one-way quantum secure direct communication system, the STIKE protocol interaction of the quantum channel and the one-time-key encryption transmission of the classical auxiliary channel both require the completion of encryption processing through security keys. The test results show that the one-way quantum secure direct communication transmission rate, security key rate, and VoIP data packet transmission rate all meet the key quantity constraints of Equations (6) and (7), thereby enabling further estimation of the duration of duplex secure voice communication. At the same time, the transmission delay of the one-way quantum secure direct communication can ensure that the VoIP voice playback delay is less than 1 s, which can guarantee the real-time experience of voice calls.

## 4. Discussion

This system adopts an architecture of “one quantum channel + one classical channel + one-time-key encryption”, which has constructed a secure and reliable duplex voice communication system. Further research work will further optimize the system architecture, replacing the existing dual channels with quantum channels to achieve duplex voice transmission over a full quantum channel; at the same time, by leveraging the eavesdropping perception characteristics of the dual channels, the communication security performance of the system will be further enhanced, and the anti-eavesdropping ability will be improved.

The various processing methods proposed in this paper (including system stable operation processing methods, real-time temperature drift compensation algorithms, and parallel multi-task processing methods, etc.) can be directly transferred and applied to the next stage of research on the full quantum channel system. Further optimization will be carried out based on the existing methods, while strictly ensuring the security of communication, continuously improving the performance indicators of the system to better meet the requirements of practical application scenarios, and promoting the practical process of one-way quantum secure direct communication technology.

## 5. Conclusions

This paper elaborates on the system composition and working principle of duplex voice communication in a one-way quantum secure direct communication system, proposes processing methods and real-time temperature drift compensation algorithms to ensure the stable operation of the system, and adopts a parallel multi-tasking processing approach to implement the STIKE protocol in view of the limited computing resources of the system. This not only improves the performance indicators of the one-way quantum secure direct communication system but also takes into account communication security and user experience. A single-way quantum secure direct communication duplex voice communication experimental platform was built to verify and test the proposed methods. The test results show that the quantum bit error rate of the system during duplex voice communication is stable at around 1.7%, and the system key generation quantity can meet the key consumption requirements of the STIKE protocol and one-time one-key encryption communication; through parallel multi-tasking processing optimization, the transmission rate of the one-way quantum secure direct communication can reach 56 kbps, the security key rate is approximately 51 kbps, and the transmission delay is reduced to around 365 ms. The above experimental data verifies the effectiveness of the proposed scheme and processing methods.

This implementation scheme fully confirms the security and real-time performance of the one-way quantum secure direct communication system in the duplex voice communication scenario. The related feasibility verification work has laid a good foundation for the practical application of quantum secure direct communication.

## Data Availability

No new data was created or analyzed in this study. Data sharing is applicable to this article.

## Abbreviations

The following abbreviations are used in this manuscript:

QSDC	Quantum Secure Direct Communication
QSDCT	Quantum Secure Direct Communication Transmitter
QSDCR	Quantum Secure Direct Communication Receiver
QBER	Quantum Bit Error Rate

## References

[1] Long G.L., Liu X.S. (2002). Theoretically efficient high-capacity quantum-key-distribution scheme. Phys. Rev. A.

[2] Deng F.-G., Long G.-L. (2004). Secure direct communication with a quantum one-time pad. Phys. Rev. A.

[3] Long G.L., Zhang H. (2021). Drastic increase of channel capacity in quantum secure direct communication using masking. Sci. Bull..

[4] Pirandola S., Braunstein S.L., Lloyd S., Mancini S. (2009). Confidential direct communications: A quantum approach using continuous variables. IEEE J. Sel. Top. Quantum Electron..

[5] Hu J.Y., Yu B., Jing M.Y., Xiao L.T., Jia S.T., Qin G.Q., Long G.-L. (2016). Experimental quantum secure direct communication with single photons. Light Sci. Appl..

[6] Zhang W., Ding D.S., Sheng Y.B., Zhou L., Shi B.S., Guo G.C. (2017). Quantum Secure Direct Communication with Quantum Memory. Phys. Rev. Lett..

[7] Zhu F., Zhang W., Sheng Y.B., Huang Y.D. (2017). Experimental long-distance quantum secure direct communication. Sci. Bull..

[8] Qi R., Sun Z., Lin Z., Niu P., Hao W., Song L., Huang Q., Gao J., Yin L., Long G.L. (2019). Implementation and security analysis of practical quantum secure direct communication. Light Sci. Appl..

[9] Chai G., Cao Z.W., Liu W.Q., Zhang M.H., Liang K.X., Peng J.Y. (2019). Novel continuous-variable quantum secure direct communication and its security analysis. Laser Phys. Lett..

[10] Cao Z.W., Wang L., Liang K.X., Chai G., Peng J.Y. (2021). Continuous-Variable Quantum Secure Direct Communication Based on Gaussian Mapping. Phys. Rev. Appl..

[11] Wang C. (2021). Quantum secure direct communication: Intersection of communication and cryptography. Fundam. Res..

[12] Zhang H.R., Sun Z., Qi R.Y., Yin L.G., Long G.-L., Lu J.H. (2022). Realization of quantum secure direct communication over 100 km fiber with time-bin and phase quantum states. Light Sci. Appl..

[13] Pan D., Song X.-T., Long G.-L. (2023). Free-space quantum secure direct communication: Basics, progress, and outlook. Adv. Devices Instrum..

[14] Pan D., Liu Y.C., Niu P., Zhang H., Zhang F., Wang M., Song X.T., Chen X., Zheng C., Long G.L. (2025). Simultaneous transmission of information and key exchange using the same photonic quantum states. Sci. Adv..

[15] Shannon C.E. (1949). Communication theory of secrecy systems. Bell Syst. Tech. J..

[16] Alvarez E., Fernandez A., Garcıa P., Jiménez J., Marcano A. (1999). New approach to chaotic encryption. Phys. Lett. A.

[17] Poppe A., Peev M., Maurhart O. (2008). Outline of the SECOQC quantum-key-distribution network in Vienna. Int. J. Quantum Inf..

[18] Song X., Zhang C., Pan D., Wang M., Guo J., Zhang F., Long G. (2023). Practical Real-Time Phase Drift Compensation Scheme for Quantum Communication Systems. Entropy.

[19] Zhang L., Wang Y., Yin Z., Chen W., Yang Y., Zhang T., Huang D., Wang S., Li F., Han Z. (2011). Real-time compensation of phase drift for phase-encoded quantum key distribution systems. Chin. Sci. Bull..

[20] Li H., Wonfor A., Weerasinghe A., Alhussein M., Gong Y., Penty R. (2022). Quantum key distribution post-processing: A heterogeneous computing perspective. 2022 IEEE 35th International System-on-Chip Conference (SOCC).

[21] Mueller R., De Lazzari C., Chirici F., Vagniluca I., Oxenløwe L.K., Forchhammer S., Zavatta A., Bacco D. (2025). Performance of Cascade and LDPC codes for information reconciliation on industrial quantum key distribution systems. IET Quantum Commun..

[22] Tang B.Y., Liu B., Zhai Y.P., Wu C.Q., Yu W.R. (2019). High-speed and large-scale privacy amplification scheme for quantum key distribution. Sci. Rep..

[23] Kleijn W.B. (1993). Encoding speech using prototype waveforms. IEEE Trans. Speech Audio Proceeding A Publ. IEEE Signal Process. Soc..

[24] Kaniewska M., Ragot S., Liu Z., Miao L., Zhang X., Gibbs J., Eksler V. (2015). Enhanced AMR-WB bandwidth extension in 3GPP EVS codec. 2015 IEEE Global Conference on Signal and Information Processing (GlobalSIP).

[25] Quatieri T.F., Mcaulay R.J. (1987). Peech transformations based on a sinusoidal representation. IEEE Trans. Acoust. Speech Signal Process..

[26] Abhijna P.C., Sangeetha N.R., Sagar J.R., Rahul R., Gupta G. (2017). Implementation of CELP encoder using Vivado HLS. 2017 2nd IEEE International Conference on Recent Trends in Electronics, Information & Communication Technology (RTEICT).

